# Molecular elements in FGF19 and FGF21 defining KLB/FGFR activity and specificity

**DOI:** 10.1016/j.molmet.2018.05.003

**Published:** 2018-05-11

**Authors:** Archita Agrawal, Sebastian Parlee, Diego Perez-Tilve, Pengyun Li, Jia Pan, Piotr A. Mroz, Ann Maria Kruse Hansen, Birgitte Andersen, Brian Finan, Alexei Kharitonenkov, Richard D. DiMarchi

**Affiliations:** 1 Department of Chemistry, Indiana University, Bloomington, IN, 47405, USA; 2 Interdisciplinary Biochemistry Graduate Program, Indiana University, Bloomington, IN, 47405, USA; 3 Novo Nordisk Research Center Indianapolis, Indianapolis, IN, 46241, USA; 4 Metabolic Diseases Institute, Department of Internal Medicine, University of Cincinnati College of Medicine, Cincinnati, OH, USA; 5 Global Research, Novo Nordisk A/S, Novo Nordisk Park, Måløv, DK-2760, Denmark

**Keywords:** FGF19, FGF21, KLB, FGFR isoforms, FGF antagonism, Structure-activity-relationship, Alanine-scan

## Abstract

**Objective:**

To signal, FGF19 and FGF21 require co-receptor βKlotho (KLB) to act in concert with FGF receptors, and yet there is appreciable variance in the C-terminal sequences of these two novel metabolic hormones where binding is believed to be primary. We seek to determine the functional consequences for these amino acid differences and determine whether such information can be used to design high potency antagonists and agonists.

**Methods:**

We employed a functional *in vitro* assay to identify C-terminal protein fragments capable of fully blocking KLB-mediated FGF19 and 21 receptor signaling. The key residues in each hormone responsible for support full bioactivity were identified through peptide-based Ala-scanning. Chemical optimization of the peptides was employed to increase their antagonistic potency. An optimized sequence as a substituted part of a full length FGF21 was assessed for enhanced FGFR/KLB-mediated agonism using tissue culture and obese mice.

**Results:**

C-terminal FGF19 and FGF21 peptides of relatively short length were observed to potently inhibit the activity of these two hormones, *in vitro* and *in vivo*. These FGFs of different sequence also demonstrated a striking conservation of structural determinants to maintain KLB binding. A single C-terminal amino acid in FGF19 was observed to modulate relative activity through FGFR1 and FGFR4. The substitution of native FGF21 C-terminal sequence with a peptide optimized for the highest antagonistic activity resulted in significantly enhanced FGF potency, as measured by *in vitro* signaling and improvements in metabolic outcomes in diet-induced obese mice.

**Conclusions:**

We report here the ability of short C-terminal peptides to bind KLB and function as antagonists of FGF19 and 21 actions. These proteins maintain high conservation of sequence in those residues central to KLB binding. An FGF21 chimeric protein possessing an optimized C-terminal sequence proved to be a super-agonist in delivery of beneficial metabolic effects in obese mice.

## Highlights

• Short C-terminal FGF19 and FGF21 peptides inhibit FGF19 and 21 signaling.
• Integration of high potency C-terminal peptide antagonists to achieve super-agonism in full-length proteins.
• Modulation of FGFR-isoform specificity by the C-terminal lysine of FGF19.
• Superior efficacy of FGF analogs optimized via KLB binding.

## Graphical abstract

**Figure:**
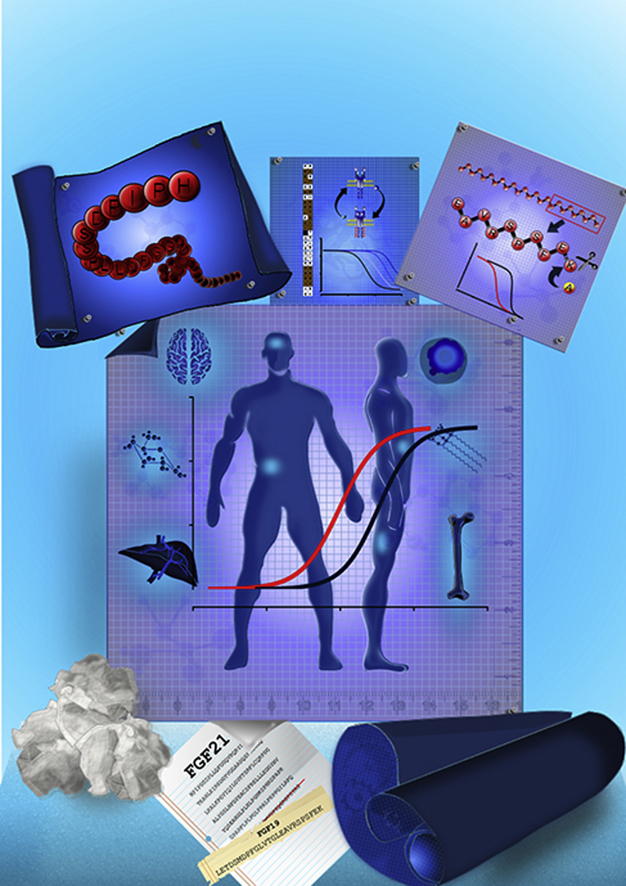

## 1 Introduction

Fibroblast growth factors 19 and 21 (FGF19 and FGF21) structurally belong to the FGF superfamily. In contrast to the classical FGFs that function in an autocrine/paracrine manner, these proteins have endocrine capabilities due to their low affinity for heparan-sulfate proteoglycans [1], [2], [3], [4]. Biochemically, they both require transmembrane β-Klotho (KLB) as a co-factor to facilitate signaling through various FGF receptors (FGFRs) [5], [6], [7], [8] and display overlapping metabolic pharmacology in rodents [9], [10], [11]. However, FGF19 is a potent inducer of hepatocellular carcinomas in mice [12], whereas FGF21 is not [13], [14].

Structurally, the termini of FGF21 are critical for effective signaling. Progressive amino acid truncation at either end of the protein sequentially impairs FGF21 bioactivity, but via different mechanisms. Shortening at the N-terminus weakens its ability to activate the FGFR/KLB-complex but does not impede binding to KLB. In contrast, consecutive C-terminal shortening diminishes FGF21 interaction with KLB to eventually eliminate the receptor-complex engagement [15], [16]. The importance of these findings is underscored by the report that Fibroblast Activation Protein (FAP) degrades both termini to inactivate FGF21, and as such has been implicated in the pathophysiology of metabolic disease [17], [18], [19], [20]. The basis for receptor activation by FGF19 is generally believed to align with what has been established for FGF21. The N-terminal region of FGF19 influences FGFR specificity [21], [22] while its C-terminal part is involved in KLB binding [23]. The FGF21 variant lacking seventeen N-terminal amino acids (FGF21^18−181^, also known as ΔN17) is capable of antagonizing the actions of both hormones [9], [24], but the structural elements for KLB recognition by at least FGF21 are only recently being explored [25]. Curiously, the sequence identity in the C-terminal region of the two proteins is less than 40%, which is seemingly low for a common binding site.

FGF19 and FGF21 effectively correct metabolic abnormalities in rodents, including regulation of glucose homeostasis [11], [13], [26], [27], [28]. In humans, FGF21 agonists improve dyslipidemia, insulin resistance, and body weight, while such studies for FGF19 agonists have yet to be communicated. Unexpectedly, no meaningful improvement of blood glucose was observed with FGF21 treatment in diabetic patients [29], [30], possibly due to insufficient dose intensity. Indeed, dose-proportional glucose lowering was observed in diabetic, non-human primates in an acute manner but only at supra-pharmacologic levels, beyond those clinically tested [31], [32]. It is thus plausible that FGF21 super-agonists might successfully reverse clinical hyperglycemia. In this regard, a protease-stabilized FGF21 analog improved blood glucose in obese, non-diabetic monkeys at doses where native FGF21 failed [33].

We sought to determine if there is a common basis to KLB binding for the C-terminal segments of FGF19 and FGF21, since these proteins are as different in amino acid sequence as they are alike. Such knowledge could be of great importance in achieving enhanced KLB affinity that might enable super-agonism. We began our investigation by determining the minimal fraction of FGF21^18−181^ necessary to fully antagonize FGF21 function. The recognition that the C-terminal 25 residues of FGF21 antagonized FGF19 and FGF21 activity substantially simplified the evaluation of the structural elements defining KLB binding in the full-length proteins, which are more than 180 amino acids in size. A full alanine scan (Ala-scan) of FGF19- and FGF21-based peptides identified specific amino acids essential to receptor activation, and the antagonism in cellular assays closely associated with binding affinity to the FGFR1/KLB complex in a cell-free system. The impact of specific alanine substitutions in antagonism correlated with changes in agonism when introduced to full-length FGF21. *In vivo*, the most potent FGF19-based peptide antagonist attenuated FGF19 and FGF21 signaling in adipose tissue and pancreas, and a FGF21-19A hybrid protein revealed improved metabolic efficacy in mice relative to native FGFs. Collectively, our results map the KLB binding elements in FGF19 and FGF21 at single amino acid resolution and specify a common functional signature by which these endocrine hormones signal via FGFR/KLB complexes, despite the appreciable difference in their native sequences.

## 2 Materials and methods

### 2.1 Peptide synthesis

FGF21 and FGF19 C-terminal peptides were prepared as C-terminal amides by Fmoc solid-phase methodology on H-Rink-Amide-ChemMatrix resin (PCAS BioMatrix Inc) with DIC/6-Cl-HOBt (N,N′-Diisopropylcarbodiimide (Sigma Aldrich), 1-Hydroxy-6-chloro-benzotriazole (Aapptec) activation employing CS336X (CSBio) or Symphony (Gyros protein technologies) automated peptide synthesizer. All amino acid residues were purchased from Midwest Biotech. Peptides were cleaved from the resin and side chain protection was removed by treatment with trifluoroacetic acid (TFA) containing 2.5% triisopropylsilane (TIS) and 2.5% H_2_O as scavengers for two hours. For FGF19 peptides the scavenger mixture was extended to contain 1% methyl sulfide and 1% 2, 2’-(ethylenedioxy)diethanethiol. Following precipitation in ethyl ether, the peptides were solubilized in 20% aqueous acetonitrile (ACN) and lyophilized. Analogs were purified by preparative reverse phase chromatography with Waters HPLC system using a Kinetex C8 column (5 μm, 100 Å, AXIA-Packed LC Column 250 × 21.2 mm; Phenomenex). A linear gradient of increasing ACN in aqueous 0.1% TFA was employed over 90 min.

### 2.2 Protein synthesis

Human FGF21 (UniProtKB #Q9NSA1 amino acid sequence: 29–209 and here designated as 1–181), FGF19 (#O95750: 23–216 here designated as 1–194), FGF21^18−181^ (FGF21 46–209 here designated as 18–181), FGF21-19A (FGF21 29–184 fused with FGF19 191–216 (K216A)), here designated as FGF21 1–156 and FGF19 169–194 (K194A), FGF23 (#Q9GZV9: 25–251), FGF1 (#P05230: 16–155 here designated as 1–140), FGF1HD (here designated as FGF1-140, Q40P, S47I, H93G, K112N, K118E [50], or FGF2 (#P09038: 143–288) cDNA sequences gene sequences (Integrated DNA Technologies) were individually inserted in LIC-SUMO vector, which is a modified expression pET21b vector (Novagen) containing yeast small ubiquitin-like modifier (SUMO) sequence followed by 6xHis tag and deletion of MCS region, using In-Fusion HD EcoDry Cloning Plus kit (Clontech). For the generation of point mutant analogs, the corresponding primers were designed (Integrated DNA technologies) and mutagenesis was performed by standard PCR method using the desired LIC-SUMO plasmid as template. Stellar *E. coli* competent cells (Clontech) were transformed to amplify the plasmid, which was harvested with QIA Miniprep kit (Qiagen). The positive clones isolated by LB/Ampicillin agar plate selection were confirmed by DNA sequencing and OrigamiB (DE3) *E.coli* cells (Novagen) for FGF21/19/23 analogs and BL21 (DE3) *E. coli* cells (Novagen) for FGF1/2 analogs were transformed for protein expression. Thereafter the cells were cultured to 0.6–0.8 OD600 at 37° C and induced by 0.2 mM isopropyl-D-thiogalactoside (IPTG) at 25° C overnight. The cells were harvested, lysed by sonication, and protein was enriched by Ni-NTA column (Qiagen). Imidazole (500 mM) containing Tris buffer was used to elute the protein which was subsequently digested with SUMO protease ubiquitin-like-specific protease 1 and pure FGF19/21/23 analogs were obtained by affinity chromatography with Q-Sepharose (GE Healthcare), or FGF1 and FGF2 analogs by SP-Sepharose (GE Healthcare) using fast flow liquid chromatography technology (GE Healthcare).

### 2.3 Purity and concentration estimation of the peptides and proteins

The purity of the biosynthesized proteins and chemically synthesized peptides was assessed by LC-MS (Agilent 1260 Infinity-6120 quadrupole mass spectrometer). All preparations were analyzed by reverse-phase Kinetex C8 column (2.6 μm, 100 Å, LC Column 75 × 2.1 mm; Phenomenex) with a linear gradient of 10–80% ACN over 10 min at a flow rate of 1.0 ml/min using aqueous 0.05% TFA and aqueous 0.05% TFA/90% ACN elution buffers. All proteins and FGF21 peptides were obtained to >90% purity. The purity within the set of 19C26 Ala-scan peptides was assessed by their 214 nm absorbance LC profile, and the concentrations were appropriately adjusted for the *in vitro* assays to reflect the concentration of the desired peptide in the preparation. The concentration of each protein and peptide was determined based on their UV absorbance on NanoDrop spectrophotometer (ThermoFisher Scientific), and the extinction coefficients for respective sequences were obtained with online tools (Prot pi peptide or ExPASy ProtParam). FGF21, FGF19, FGF21-19A protein preparations used for *in vivo* studies were purified from endotoxin by ToxinEraser Endotoxin removal kit (GenScript) to ensure 0.5 EU/mg or less endotoxin.

### 2.4 Cell lines

For generating the 293T HEK cell line with stable expression of co-receptors (293/KLB), the human β-Klotho (KLB) (GenScript) or human Klotho (KL) (GenScript) gene was subcloned into pcDNA3.1/Zeocin resistance vector (Invitrogen) by NheI and NotI restriction enzyme sites. The cells were transiently transfected at 80% confluency using Lipofectamine 3000 (Invitrogen). Selection for KLB/KL-expressing cells was initiated 48 h post-transfection in the growth media containing 100 μg/ml of Zeocin (Gibco) and continued for 4 weeks with fresh media added every third day. KLB and KL expression in pooled cells was confirmed by Western blot and a functional pERK AlphaLISA assay.

For generating receptor specific BaF3 cell line, human FGFR1c cDNA was cloned into pMXs-IRES-Puro vector (Cell Biolabs) to generate the expression plasmid pMX-FGFR1c-IRES-Puro. Human FGFR4 sequence was re-engineered to enhance signaling by constructing chimeric cDNA encoding the extracellular region of FGFR4 fused to the intracellular region and tyrosine kinase domain of FGFR1c [56], and the chimeric sequence was cloned into pMX-IRES-Puro vector to generate the expression plasmid pMX-chimeric FGFR4-1c-IRES-Puro. Human KLB cDNA was cloned into pMXs-IRES-Neo vector (Cell Biolabs) to generate expression plasmid pMX-KLB-IRES-Neo. BaF3 cells were transfected with pMX-FGFR1c-IRES-Puro and pMX- FGFR4-1c chimera-IRES-Puro expression plasmids respectively by electroporation (Xcell machine, Bio-Rad, 950 μF, 370V). Cells were selected with media containing 1 μg/ml Puromycin for 10–12 days. After characterization, the positive cells were further transfected with pMX-KLB-IRES-Neo with the same method. Cells were selected with media containing 1 μg/ml Puromycin and 1 mg/ml Geneticin for 10–12 days. After characterization, the positive cells co-expressing human FGFR1c/KLB or chimeric FGFR4-1c/KLB respectively were scaled up for use. 293T HEK (human embryonic kidney) cells (ATCC) and Hep3B (human hepatocellular carcinoma) cells (ATCC) were cultured in DMEM High glucose GlutaMAX (Gibco) supplemented with 10% Fetal Bovine Serum (Gibco) and 1x Antibiotic-Antimycotic (Gibco) at 37° C, 95% humidity and 5% CO2. BaF3 (murine lymphoid) cells obtained from ATCC were cultured in RPMI1640 (Gibco), supplemented with 10% heat inactivated Fetal Serum (Biochrom AG), 0.5 ng/ml Interleukin-3 (Sigma Aldrich), 1 μg/ml Puromycin (Gibco), 1 mg/ml Geneticin (Gibco), and 100 U/ml Penicillin-Streptomycin (Gibco). Cells were cultured at 37° C in a humidified atmosphere with 5% CO2.

### 2.5 pERK AlphaLISA assay

293T HEK cells expressing KLB (or KL) or Hep3B cells were plated to 5–6 x 10^4^ cells/well density in 96-well cell culture plate coated with poly-d-Lysine (Corning). The cells were serum starved for four hours in 0.1% BSA fraction V (ThermoFisher) containing media prior to treatment with protein and/or an antagonist peptide at the mentioned concentrations for 10 min at 37° C. For the antagonist assays, a chosen fixed dose within the range of 0.8–30 nM (depending on the cell type) of FGF21 or FGF19 stimulant was added to all the wells. BaF3 cells expressing human KLB/FGFR1c or human KLB/FGFR4-1c chimera were seeded at 5 × 10^5^ cells/well density in 96-well cell culture plate and serum-starved overnight. FGF21/19 analogs were diluted in 0.02% Tween-20 containing RPMI1640 medium and the cells were treated for 15 min at 37° C. Following the respective cell treatments, the cells were lysed (with lysis buffer provided in the kit) for 10 min at room temperature on a shaker and the lysate was mixed with reaction mixture (consisting of reaction buffer, activation buffer, acceptor and donor beads provided in the kit). Phosphorylated ERK1/2 (pERK) levels were subsequently recorded by EnSpire Alpha or Envision Multilabel plate reader (Perkin Elmer). The cell assays corresponding to 293/KLB or Hep3B cells were carried out with AlphaLISA SureFire Ultra pERK1/2 (Thr 202/Tyr204) kit (Perkin Elmer), and BaF3 cells were done using AlphaScreen pERK1/2 (Thr202/Tyr204) kit (Perkin Elmer).

### 2.6 Binding assay

Biotinylated FGF21 was coupled to Streptavidin-coated donor beads (AlphaScreen technology, Perkin Elmer), and the ectodomains of human FGFR4 fused to Fc (R&D Systems) or FGFR1c fused to Fc (R&D Systems) were coupled to Protein A acceptor beads (AlphaScreen technology, Perkin Elmer). Human KLB protein (R&D Systems) was added to the mixture of biotinylated-FGF21-Streptavidin donor beads and FGFR-Fc-Protein A acceptor beads, bringing the beads in close proximity producing a signal, in the presence of 5 μg/ml Heparin and 0.1% BSA. Increasing doses of FGF21 C-terminal peptides were added and if their binding to the receptor complex were to take place, the biotinylated FGF21 would be displaced to consequently decrease the signal. The EnVision Multilabel Plate Reader (Perkin Elmer) was used to quantify the emitted light signal at 570 nm.

### 2.7 Cellular signaling data analysis

All cellular phosphorylation data were analyzed using 3-parameter non-linear regression curve in GraphPad Prism 7. Graphs are represented as relative maximal (100%) response of the positive control. All other test analog signals were adjusted accordingly as % values. For antagonistic assays, the difference between the p-ERK signal at baseline (fixed dose of FGF-stimulation, no antagonist peptide added) and the highest dose of the positive control, either FGF21^18−181^, 21C25 or 19C26 for respective assay, was set to 100%. Similarly, for an agonist assay, the difference between the p-ERK signal of baseline (only cell culture medium, no agonist) and the highest dose of the positive control either FGF21 in 293/KLB cells or FGF19 in Hep3B cells was used to establish 100% response. Each peptide and protein analog was tested in triplicates in three independent experiments (n = 3), and normalized graphs plotted are expressed as mean ± SD unless otherwise noted.

### 2.8 Regression analysis

**Figure 1:**
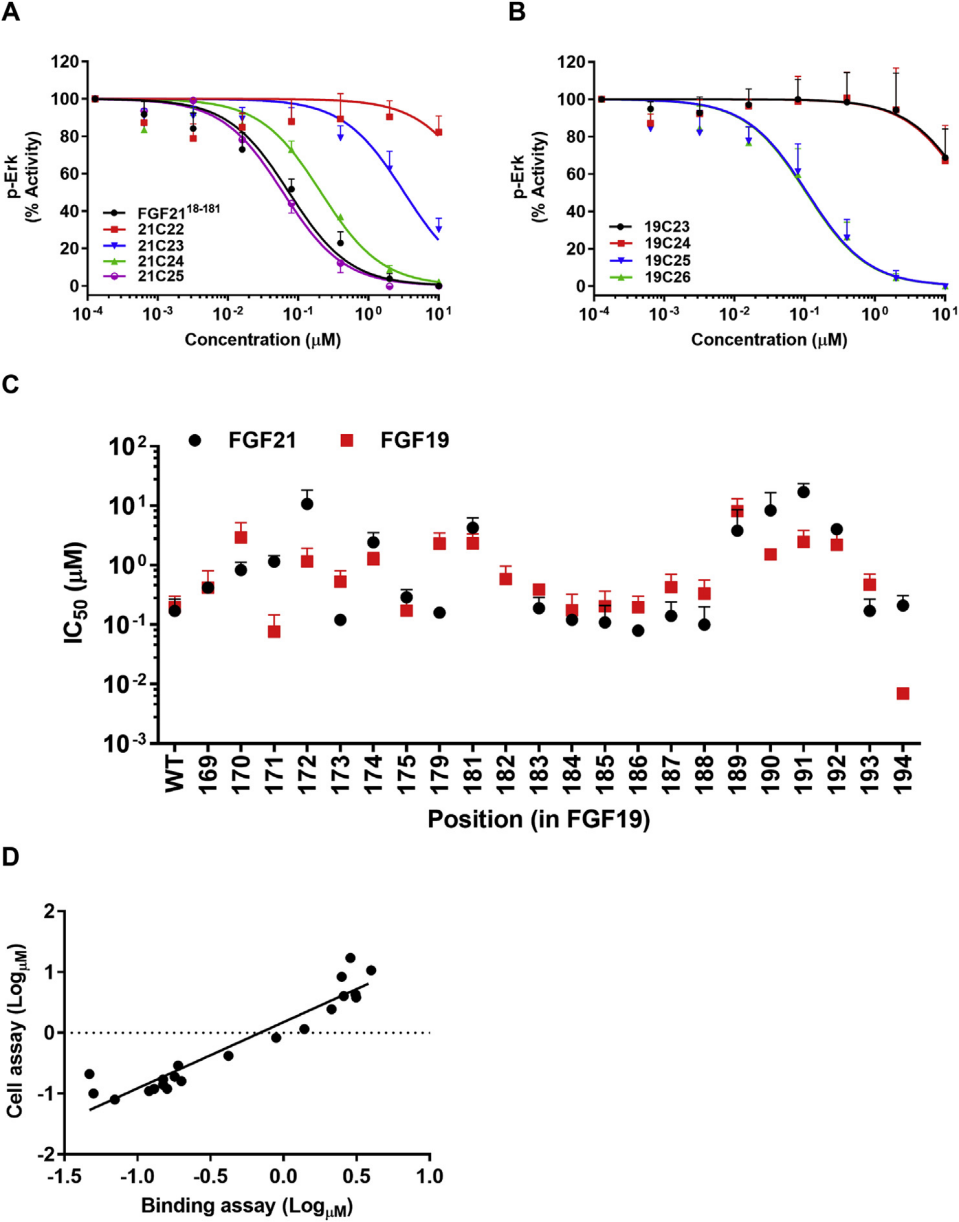
**C-terminal FGF21 and FGF19 peptides inhibit FGF21 signaling and receptor binding.** (A and B) Activity of C-terminal peptide antagonists to inhibit FGF21 signaling in 293/KLB cells. (A) FGF21 peptides 21C22 (red), 21C23 (blue), 21C24 (green), 21C25 (purple), and the reported antagonist FGF21^18−181^ (ΔN17; black) were tested; (B) FGF19 peptides 19C23 (black), 19C24 (red), 19C25 (blue), 19C26 (green) were tested. The graphs show representative curves plotted by normalizing the pERK signal of each analog with the native peptide's response (A) 21C25 or (B) 19C26 respectively, mean ± SD, n = 3. (C) Antagonistic potencies (IC_50_ values, Y axis) of 21C25 Ala-peptides (FGF21, black) and 19C26 Ala-peptides (FGF19, red) in blocking FGF21 signaling were plotted with corresponding FGF19 position numbering referenced on the X-axis (inactive Ala-mutants are excluded, see Table 1 for FGF21-FGF19 sequence alignment). (D) Regression analysis (r^2^ = 0.90) of the log-transformed IC_50_ values of 21C25 Ala-scan peptides in cell signaling assay (Y-axis, mean, n = 3) relative to their competitive displacement of FGF21 binding from soluble human FGFR1c/KLB complex (X-axis, mean, n = 2).

The correlation between the average IC_50_ values of FGF21 Ala-scan peptides measured by cell signaling (mean, n = 3) and binding studies (mean, n = 2) was calculated by linear regression (r^2^ = 0.62). To control for outliers, each data set was log-transformed and subsequently analyzed by linear regression (r^2^ = 0.90) was obtained. The log-transformed plot is presented in Figure 1D.

### 2.9 Animals

All animal studies were approved by and performed according to the guidelines of the Institutional Animal Care and Use Committee of the University of Cincinnati (Cincinnati, USA). Eight-week old male C57BL/6J mice (Jackson Laboratories) were given ad libitum access to either a chow diet (lean mice), or a high-fat high-sugar diet (HFD, DIO mice) containing 58% kcal from fat (Research Diets, catalog# D12331). For DIO mice, animals were maintained on the HFD for a minimum of 3 months prior to initiation of the pharmacological studies. The mice were maintained at 22 °C on a 12-h light–dark cycle with free access to water. Before the beginning of the studies, mice were randomized into treatment groups according to body weight. All injections and tests were performed during the light cycle with a group size of n = 8.

### 2.10 *In vivo* peptide antagonism study

Mice were administered vehicle, FGF21 (1 mg/kg), or FGF19 (1 mg/kg) alone or in combination with 19C26,A^26^ (30 mg/kg), (dosed 15 min prior to FGF21 or FGF19 administration) via *sc* injections. One-hour post FGF21 or FGF19 dosing, the mice were sacrificed and their plasma, pancreas, and epididymal white adipose tissue (eWAT) were harvested for analyses. Total RNA was isolated from pancreatic and eWAT tissues using Trizol phenol-chloroform extraction (Invitrogen, Cat#155596026) and purified using an RNeasy mini (Cat#74104) and on-column DNAse digest (Cat#79254) kits (Qiagen). Following reverse transcription, levels of *cFos* and early growth response-1 (*EGR1*) mRNA levels were subsequently probed by quantitative PCR (ThermoFisher) and analyzed by ΔΔCT method.

### 2.11 *In vivo* FGF21 agonism study

Mice were administered vehicle, FGF21 or FGF21-19A (0.1, 0.3 and 1 mg/kg) daily via *sc* injections and their body weight measurements were made on alternative days. At the end of 7 day treatment, blood was collected from tail veins using EDTA-coated microvette tubes (Sarstedt), immediately placed on ice, centrifuged at 5000 × g and 4 °C for 10 min, and plasma was stored at −80 °C until analyzed. Triglycerides and cholesterol levels were analyzed using colorimetric assay (Thermo Fisher).

### 2.12 *In vivo* data analysis

Statistical analyses for *in vivo* experiments were performed on data using a one- (Figure 5A,B and 5D) or two-way (Figure 5C) ANOVA followed by Tukey post hoc multiple comparison analysis. All results are presented as mean ± SEM, n = 5–8, and P < 0.05 was considered significant. Group size estimations were based upon a power calculation to minimally yield an 80% chance to detect a significant difference in body weight of P < 0.05.

## 3 Results

### 3.1 Short C-terminal peptides are competitive antagonists of FGF19 and FGF21 signaling

The C-terminal region of FGF21 binds to KLB as previously implied by FGF21^18−181^ antagonism [9]. However, this N-terminally shortened form represents approximately 90% of the native protein, with the specific molecular elements responsible for antagonism undefined. We chemically synthesized peptides of varying length derived from the C-termini of FGF19 and FGF21 to analyze their relative ability to inhibit signaling of these endocrine FGFs. For comparative purposes, we utilized an *in vitro* assay in which we monitored dose-dependent attenuation of FGF-induced ERK phosphorylation in human KLB overexpressing 293T HEK cells (293/KLB).

C-terminal FGF21 peptides of 29 and 38 residues blocked FGF21 signaling with efficacy and molar potency comparable to what had previously been observed for FGF21^18−181^ (data not shown). Further shortening revealed that the C-terminal 25-residue peptide (21C25, see Table S1 for abbreviations) retains complete inhibitory behavior (Figure 1A). Additional truncations by single amino acids progressively reduced potency to a point where peptides of 22 residues (21C22, Figure 1A) or fewer (data not shown) had no effect within the dose range examined. Analogous results were obtained in study of FGF19-based peptides where compounds of 24 amino acids (19C24) or less were inactive, while the peptides of 25 and 26 amino acids (19C25 and 19C26) were comparable to FGF21^18−181^ in their antagonistic effect (Figure 1B). We thus concluded that to antagonize signaling of each parent endocrine factor with maximal efficacy, 25 amino acids is the minimal length of FGF19 and FGF21 C-terminal peptides. Nonetheless, for sequence alignment considerations, all future experiments utilized FGF19-based peptides that were 26 amino acids in size (19C26). It contains a single insertion of threonine at position 182 (Table 1) compared to the FGF21 sequence, which was shown to be unnecessary for antagonistic efficacy, as its deletion (19C26,ΔT^14^) was observed to have only a negligible effect (Fig. S1A).

**Table 1:** *In vitro* antagonistic activities of C-terminal FGF21 and FGF19 Ala-scan peptides.

IC_50_ values (presented in μM, mean ± SD, n=3) and fold-changes in antagonistic activity of Ala-scan mutants *vs.* respective native peptides determined by inhibition of FGF21-induced pERK signaling in 293/KLB cells. The peptide sequences are aligned to maximize sequence identity (157-181 in FGF21, *vs.* 169-194 in FGF19) with native amino acids shown in one letter code. The alanine substitutions of significantly lower potency compared to native peptide (4x or more) are highlighted in red bold font. NC denotes where IC_50_ values were Not Calculated due to negligible activity at the tested concentrations.

21C25 and 19C26 also blocked FGF19 activity comparably to that of FGF21 in 293/KLB and Hep3B cells (Figs. S1B and S2B). However, both of these molecules were unable to attenuate signaling of FGF1 in 293/KLB cells, or FGF23 in 293/KL cells overexpressing the FGF23 co-factor human Klotho [34] (data not shown), confirming their KLB-dependent character. Thus, C-terminal peptides of as little as 15% the length of native FGF19 and FGF21 can potently antagonize signaling of these endocrine hormones.

### 3.2 Defining structure-activity relationship in the C-terminal peptides

To identify the individual amino acids that are critical in mediating the KLB interaction, complete alanine scans for 21C25 and 19C26 peptides were conducted. The degree of antagonism within these two sets of mutants with single-site alanine substitutions varied profoundly but could be grouped in two common categories. Group A, consisting of thirteen peptides for FGF19 and one less for FGF21, depicts those entities that generally retained the *in vitro* performance of the native sequence, as assessed by maximal effect and inherent potency (Table 1). In contrast, Group B, consisting of thirteen mutants for each FGF (red color, bold font), represents alanine substitutions that substantially reduced or completely destroyed antagonism. Of the twenty-six C-terminal amino acids studied, there are only ten sites (<40%) that are identical among the two endocrine FGFs, and each of them behaved similarly when substituted with alanine. Seven of these ten residues demonstrated a sizable change in activity when substituted (Group B), consistent with common logic that sequence identity confers biological importance. Separately, there were three sequence-aligned sites in FGF21/FGF19 that differed in their response to alanine substitution, D159/E171, S167/G179 and S181/K194, respectively (Table 1, Figure 1C). The first of these is unexpected since it constitutes a conservative substitution of one acidic amino acid for another. The second change might result from secondary structure alteration, as glycine is capable of supporting turns not typically seen with serine. The last one represents a non-conservative change and is worthy of additional consideration.

**Figure 2:**
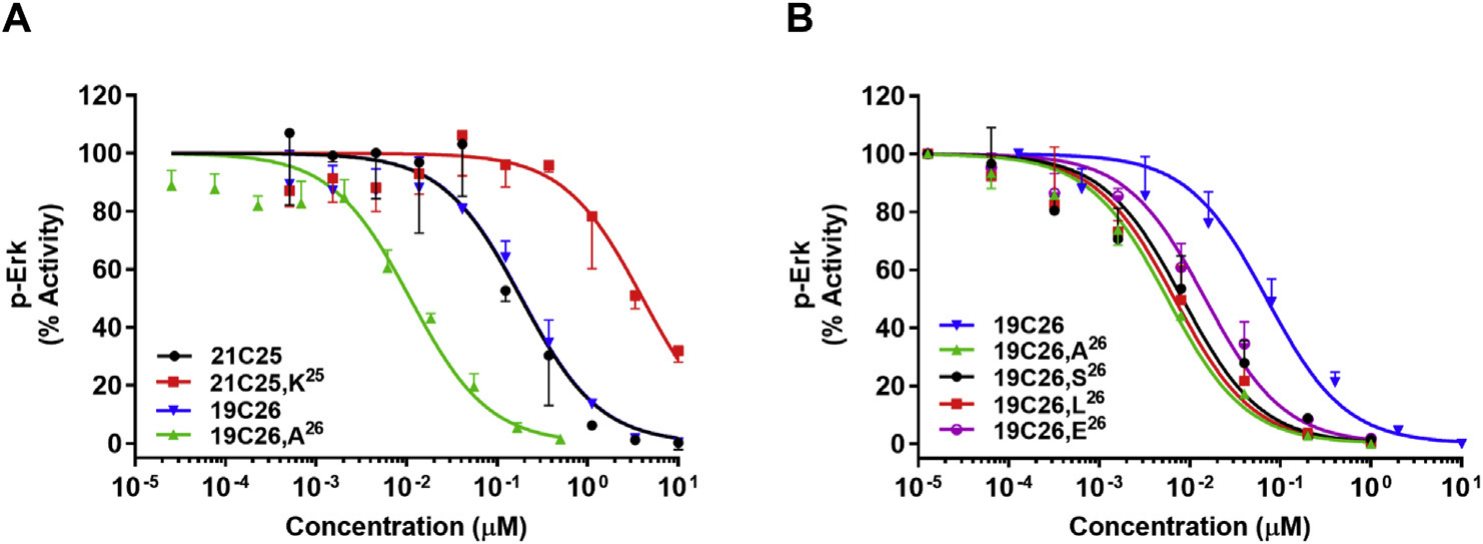
**The effect of the C-terminal residue in peptides on FGF21-antagonism in 293/KLB cells.** FGF21-induced pERK activity in the presence of increasing concentrations of peptide antagonists. (A) 21C25 (black), 21C25,K^25^ (red), 19C26 (blue) and 19C26,A^26^ (green); (B) 19C26 (blue), 19C26,A^26^ (green), 19C26,S^26^ (black), 19C26,L^26^ (red), 19C26,E^26^ (purple). The graphs show representative curves plotted by normalizing the pERK signal of each analog with the standard peptide's response (A) 21C25 or (B) 19C26,A^26^ respectively, mean ± SD, n = 3.

Alanine substitution of the terminal lysine in FGF19 was the only change that led to increased activity in the corresponding analog, 19C26,A^26^. Relative to the native sequences represented by 19C26, 21C25, and FGF21^18−181^, this single site alanine mutant inhibited FGF21 (Figure 2A) and FGF19 (Fig. S1B) signaling with at least twenty-fold greater potency (Table 1). The molecular rationale for this enhanced antagonism is not intuitively obvious. Conversely, when cationic lysine is introduced to 21C25 (21C25,K^25^) replacing the native serine, the inhibitory activity is significantly decreased (Figure 2A). The relative order of potency for these peptides was maintained when FGF21 (Fig. S2A) and FGF19 (Fig. S2B) antagonism was studied in non-engineered human liver Hep3B cells. To note, the super-antagonism demonstrated by 19C26,A^26^ was not specific to alanine substitution, as mutation of the terminal lysine with serine (hydrophilic), leucine (hydrophobic), or glutamic acid (anionic) all improved the antagonistic potency as compared to 19C26 (Figure 2B). Finally, to substantiate the mechanism by which these peptides inhibit FGF signaling, we evaluated their binding affinity to soluble FGFR1/KLB receptor complex in a cell-free assay (Table S2). In this assessment the 21C25 Ala-mutants competed with FGF21 to bind FGFR1c/KLB, and their relative activity in this study nicely correlated with cell-based signaling measurements (r^2^ = 0.90, Figure 1D, Table S2).

### 3.3 Mutations that alter peptide antagonism affect FGF21 and FGF19 agonism

**Figure 3:**
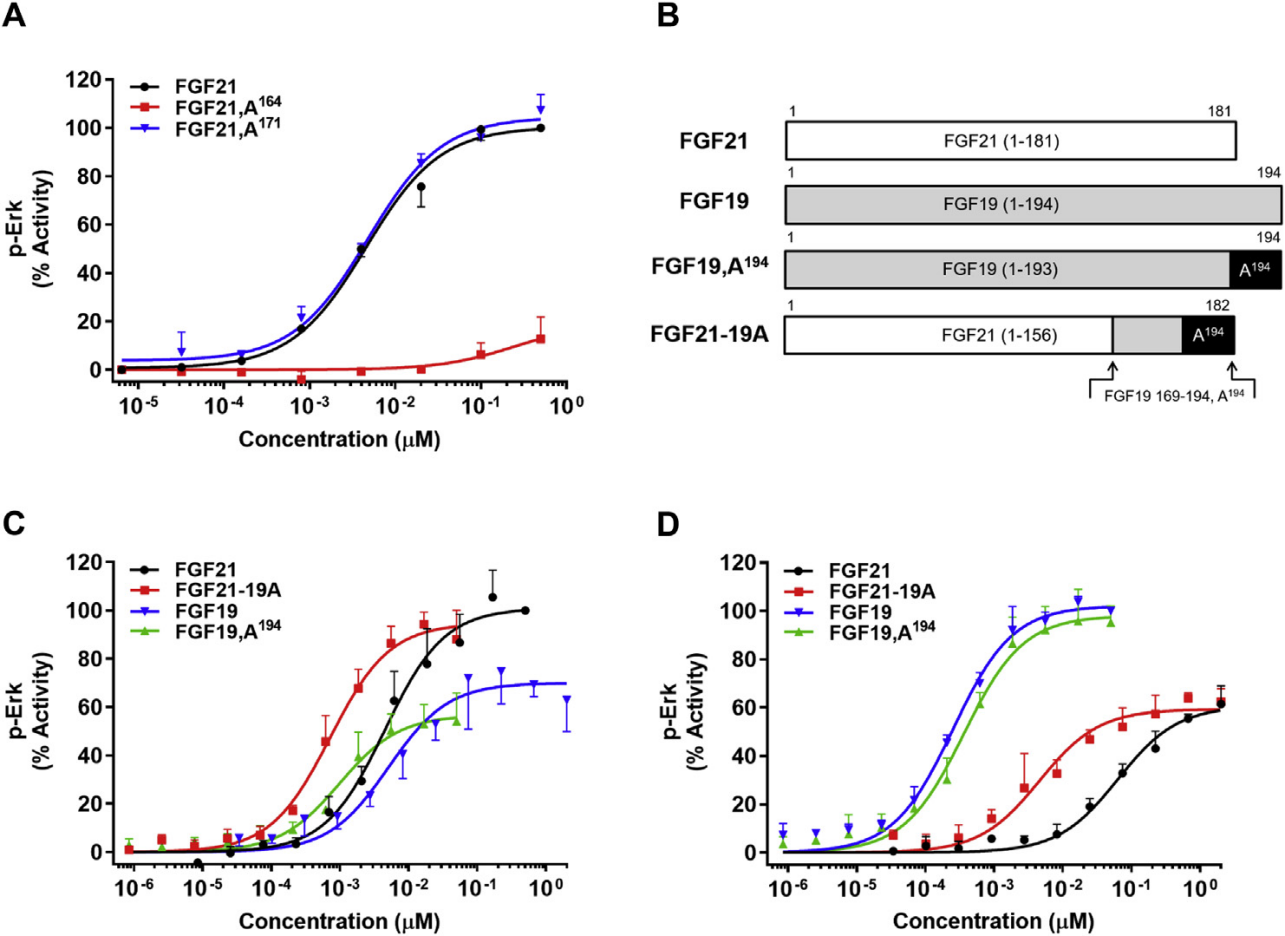
**Dose-dependent *in vitro* activity of FGF19 and FGF21 analogs.** (A) Relative pERK activity induced by FGF21 (black), FGF21,A^164^ (red), FGF21,A^171^ (blue) in 293/KLB cells; (B) Schematic showing the native FGF21 and FGF19 sequences along with the modified analogs FGF19,A^194^ and FGF21-19A which used in the subsequent studies. (C and D) Relative pERK activity induced by (C) FGF21 (black), FGF21-19A (red), FGF19 (blue), FGF19,A^194^ (green) in 293/KLB cells; (D) FGF21 (black), FGF21-19A (red), FGF19 (blue), FGF19,A^194^ (green) in Hep3B cells. The graphs show representative curves plotted by normalizing the pERK signal of each analog with the native protein's response (A and C) FGF21 (D) FGF19 respectively, mean ± SD, n = 3.

To investigate the translational impact of the peptide structure-antagonism relationship to protein-based agonism, two individual alanine substitutions were introduced to native FGF21. One change represents a deleterious D164A substitution and the other one a benign P171A mutation (Table 1). The FGF21,A^171^ analog proved nearly identical in efficacy to the wild type protein, while the FGF21,A^164^ displayed only negligible activity (Figure 3A). The conformational integrity of the inactive FGF21,A^164^ analog was assessed by circular dichroism relative to native protein (data not shown). No significant difference in composite secondary structure was observed between these two proteins suggesting that the change in bioactivity resulted from local alteration in conformation.

Given these results we explored whether the enhanced antagonistic potency recorded for 19C26,A^26^ could translate to superior protein-based agonism. To address this question, two proteins, an FGF19 analog with single C-terminal lysine substitution to alanine (FGF19,A^194^) and a FGF21-19A hybrid composed of the core FGF21 1–156 sequence followed by a C-terminal 19C26,A^26^ peptide were biosynthesized (Figure 3B). FGF21-19A proved to be functional and significantly more potent when compared to native FGF21 in 293/KLB (Figure 3C) and Hep3B cells (Figure 3D). In contrast, FGF19,A^194^ was nearly tenfold more active than FGF19 in the 293/KLB assay (Figure 3C) but was comparable when studied in Hep3B cells (Figure 3D).

**Figure 4:**
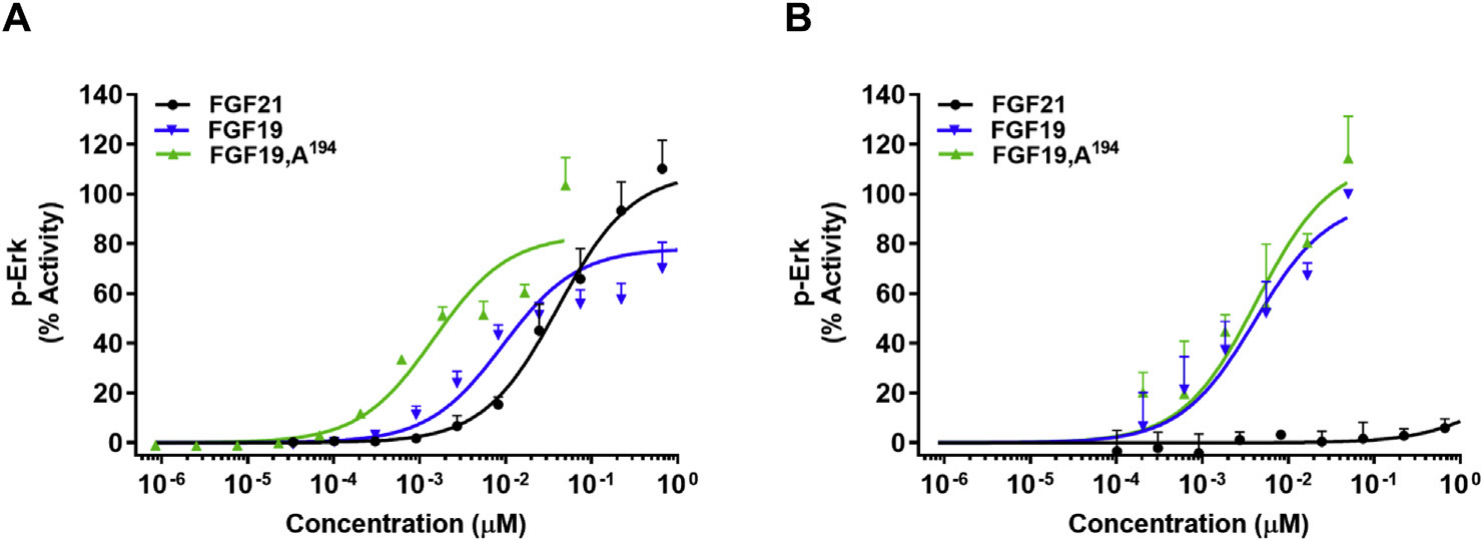
**Effect of a C-terminal alanine substitution on the activity of FGF19 in BaF3 cells co-expressing KLB with either FGFR1 or FGFR4.** Dose dependent *in vitro* activation of pERK by FGF21 (black), FGF19 (blue), FGF19,A^194^ (green) in cells expressing (A) human FGFR1/KLB; and (B) human FGFR4-1c chimera/KLB receptor complexes respectively. The graphs show representative curves plotted by normalizing the pERK signal of each analog with the native protein's response (A) FGF21 (B) FGF19 respectively, mean ± SD, n = 3.

FGF19 and FGF21 signal via different FGFR isoforms with FGFR4 being of greater importance relative to FGFR1 for FGF19 signaling while FGF21 favors FGFR1 and does not activate FGFR4 [35], [36], [37]. This bias might explain the cell-type selectivity in bioactivity observed for the FGF19,A^194^ analog when compared to the native protein as the expression profile of FGF receptors differs between 293T HEK and Hep3B cells [9]. To examine the prospect that the C-terminal lysine in FGF19 defines the FGFR isoform specificity, the bioactivity of the native FGFs and FGF19,A^194^ was assessed in BaF3 cells that co-expressed KLB with either FGFR1 or FGFR4. As expected, FGF21 was active in KLB/FGFR1 co-expressing cells (Figure 4A), while inducing minimal signaling at KLB complexed with FGFR4-1c chimeric receptor, an FGFR4 mimetic (Figure 4B). The single-site C-terminal mutation in FGF19 to alanine enhanced signaling at the FGFR1/KLB complex by approximately ten-fold (Figure 4A) but showed no change in bioactivity in FGFR4-1c/KLB cells (Figure 4B). The impact of C-terminal alanine mutation on FGF19 receptor selectivity was further assessed in the FGFR isoform-specific binding assay. Similar to what was observed in the cell-based agonism assays, FGF19,A^194^ interacted more potently with the FGFR1/KLB complex than the native proteins but was indistinguishable from FGF19 in FGFR4/KLB assay (Table 2). Once again, FGF21 showed negligible binding to FGFR4/KLB as opposed to high affinity interaction with the FGFR1/KLB complex (Table 2).

**Table 2:** Binding affinity (nM) of FGF19,A^194^ analog relative to native FGFs at human FGFR1c/KLB and human FGFR4/KLB receptor complexes.

	FGFR1c/KLB	hFGFR4/KLB
FGF21	122.8 ± 8.7	1224.3 ± 177.9
FGF19	269.6 ± 15.0	10.6 ± 0.3
FGF19,A^194^	35.7 ± 1.2	9.1 ± 0.0

The affinity of FGF21, FGF19, and FGF19,A^194^ for FGFR1c/KLB or FGFR4/KLB-receptor complexes was analyzed using the AlphaScreen technology. Purified ectodomains of human FGFR1c and FGFR4 were coupled to acceptor beads and biotinylated FGF21 was coupled to donor beads while adding human soluble KLB generated a signal. The binding affinities of the protein analogs were estimated by their ability to displace the biotinylated FGF21 from the pre-formed FGF21/FGFR-KLB ternary complex; values presented in nM, mean ± SEM, n = 3.

### 3.4 *In vitro* to *in vivo* translation

**Figure 5:**
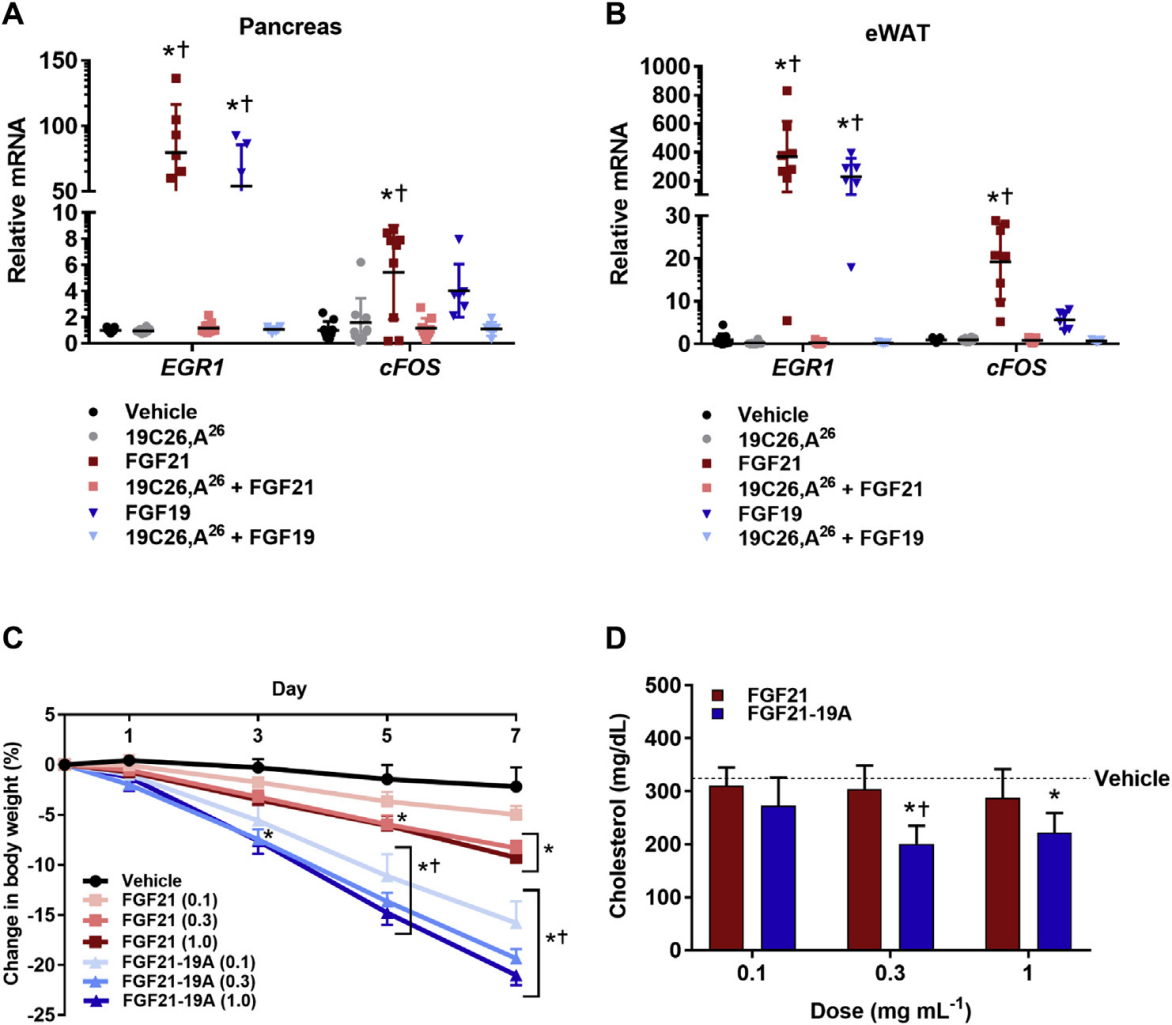
***In vivo* activities of 19C26,A**^**26**^**antagonist and FGF21**–**19A agonist in mice.** (A and B) Lean (C57BL/6J) mice were treated with either FGF19 or FGF21 (1 mg/kg) following pre-treatment with or without a peptide antagonist (30 mg/kg) as indicated. The gene induction of *EGR1* and *cFos* as measured by the relative mRNA expression was determined one hour post-treatment in (A) pancreas and (B) eWAT tissue. The results are presented as mean ± SEM, n = 5–8; vehicle (black), 19C26,A^26^ (grey), FGF21 (dark red), 19C26,A^26^ and FGF21 (light red), FGF19 (dark blue), 19C26,A^26^ and FGF19 (light blue). Each gene was analyzed by 1-way ANOVA with Tukey post-hoc analysis where statistical significance of *P < 0.05 versus vehicle and +P < 0.05 versus 19C26,A^26^ was calculated. (C and D) Dose-dependent study of FGF21-19A treatment relative to native FGF21 in DIO mice as treated daily via *sc* injection for 7 days (n = 8). (C) Change in body weight at each corresponding dose as a function of time, vehicle (black), FGF21 (0.1 mg/kg lightest red, 0.3 mg/kg red, 1.0 mg/kg dark red) and FGF21-19A (0.1 mg/kg lightest blue, 0.3 mg/kg blue, 1.0 mg/kg dark blue); data were analyzed by 2-way ANOVA with Tukey post-hoc analysis where statistical significance of +P < 0.05 was FGF21 versus FGF21-19 A at equivalent doses and *P < 0.05 versus vehicle was calculated. (D) Cholesterol levels for each corresponding dose at the end of the study, FGF21 (red) and FGF21-19A (blue), data were analyzed by 1-way ANOVA with Tukey post-hoc analysis where statistical significance of +P < 0.05 was FGF21 versus FGF21-19A and *P < 0.05 versus vehicle was calculated.

In comparable fashion to FGF21 antagonism previously reported for FGF21^18−181^, we explored the prospect that the much smaller-sized peptides might similarly function *in vivo* [9], [24]. To evaluate this possibility, FGF19 and FGF21 (1 mg/kg) were administered to mice alone or in combination with the most potent peptide antagonist 19C26,A^26^ (30 mg/kg). The degree of FGF agonism was measured one hour following subcutaneous injection by RNA expression of *cFos* and *EGR1* as transcriptional biomarkers of FGF action in white adipose tissue (WAT) and pancreas, the main target tissues for these endocrine hormones [38], [39]. As expected, FGF21 and FGF19 induced robust transcriptional activity in each tissue, and no effect on *cFos* and *EGR1* mRNA levels was observed with the peptide antagonist alone (Figure 5A,B). However, when co-dosed, 19C26,A^26^ completely eliminated the ability of FGF19 or FGF21 to stimulate gene induction (Figure 5A,B), providing evidence that peptides much smaller than FGF21^18−181^ can effectively block *in vivo* signaling of endocrine FGF. While in WAT FGFs and 19C26,A^26^ are likely acting upon adipocytes, the exact nature of the specific cell type targeted in the pancreas is unknown. In this regard, FGF21 has been reported to engage acinar cells [40], as well as pancreatic islets [41], [42].

The final objective was to translate the superior *in vitro* potency in protein agonism to enhanced metabolic pharmacology in diet-induced obese (DIO) mice. As such, the comparative efficacy of the FGF21-19A hybrid, which demonstrated ten-fold-enhanced *in vitro* potency (Figure 3C,D), was studied relative to FGF21. The obese mice were administered daily FGF proteins for a week by subcutaneous injection, at doses of 0.1, 0.3 or 1 mg/kg. FGF21 induced weight loss in a dose-dependent manner at a magnitude consistent with prior results [27], while FGF21-19A was dramatically more potent at each corresponding dose (Figure 5C). Similarly, plasma cholesterol was lowered by the protein analog (Figure 5D). Each of the FGF proteins potently reduced circulating insulin when compared to vehicle treatment. However the difference in FGF21 effect relative to the FGF21-19A did not reach statistical significance (Fig. S3A). Similarly, the comparative difference between native FGF21 and a super-agonist as assessed by fasted plasma glucose was statistically different only at the 0.3 mg/kg dose (Fig. S3B), and no change in triglycerides was observed in any treatment group (Fig. S3C). These latter observations need to be considered in the context of the FGF21 inability to further lower such measures of metabolism beyond normal levels [43] and the fact that the study was conducted in obese, non-diabetic mice with triglycerides in normal physiological range.

## 4 Discussion

The initial reports associating members of the FGF superfamily with energy homeostasis first appeared for FGF19 [10] then FGF21 [13]. Each of these factors has reduced affinity for heparan-sulfate proteoglycans [44] to enable their endocrine mode of action [45], [46], [47], as opposed to the classical FGFs that function close to site of their synthesis [48]. Furthermore, the hormone-like FGFs require a tissue-specific transmembrane co-factor KLB [49]. KLB functions as an otherwise inert cell surface protein that anchors FGF19 and FGF21 to their target tissues to facilitate signaling via FGFRs. Still, the nanomolar potencies of FGF19 and FGF21 (Figure 3A,C and 3D) are paltry relative to the picomolar activities of FGF1 and FGF2 when studied in the same assays (Figs. S4A and S4B). Furthermore, and in contrast to FGF19 and FGF21, the activity of the latter proteins is largely heparin-dependent [50] and displays bell-shape dose responses (Fig. S4C). Another endocrine protein, FGF23, is also significantly more potent than FGF21 (Fig. S4B). Consequently, the emerging perspective is that conventional FGFs, and in particular FGF1 that also signals through FGFRs, can display potent endocrine biology [51], [52], [53]. Collectively, these observations indicate that much higher FGF19 and FGF21 metabolic potency might yet be achieved via optimization of their association with the FGFR/KLB complex.

The C-terminal regions in both FGF19 and FGF21 define their KLB interaction [15], [16], [23], [25]. However, less than 40% identity in comparative alignment of C-terminal sequences for these two proteins (Table 1) suggests that their interaction with KLB may require higher-order association with other regions within these molecules. Our results reveal that relatively short C-terminal peptides of twenty-five amino acids from both of these endocrine hormones are fully sufficient to support interaction with KLB. Moreover, the limited sequence identity in the C-terminal region of FGF19 and FGF21 defines to a substantial degree the common functional elements of utmost importance to KLB binding. This was clearly demonstrated by the comparative Ala-scan peptides with eleven of the thirteen positional sites that display sizable change in activity being common between the two proteins (Table 1, Group B). The correlative manner in which Ala-substituted peptides block signaling by either hormone (Figure 1C, Table 1) also suggests that FGF19 and FGF21 utilize common KLB binding interfaces. Finally, tight association between activities assessed in signaling and cell-free binding assays (Figure 1D, Table S2) is supportive of the mechanism in peptide action occurring via direct KLB association. These results fit well with the recent report employing a biophysical approach in assessment of structural determinants that define FGF21 interaction with KLB [25]. Additionally, sequence alignment of the C-terminal sequences of FGF19 and FGF21 from numerous species (Fig. S5) reveals a high degree of conservation among the critical residues highlighted by the Ala-scan data, which fortifies the evolutionary basis of retaining important biological functions.

Alanine scanning is customarily used to identify locations where decreased activity implies structural importance. It is highly uncommon to observe a logarithmic increase in potency upon substitution with alanine [54]. Therefore, the selective increase in peptide antagonism through a single C-terminal amino acid change in 19C26,A^26^ was unexpected. Through study of additional substitutions, it is nevertheless clear that alanine is not unique in its potency-enhancing properties. Within the additional set of representative changes, only the inversely charged, anionic glutamic acid produced a subtle reduction in potency when compared to 19C26,A^26^ (Figure 2B). Furthermore, the introduction of a terminal alanine to the FGF21-based C-terminal peptide did not replicate the potency of 19C26,A^26^ (Table 1), yet replacement of the natural serine in the FGF21 peptide with lysine (21C25,K^25^) similarly reduced its antagonistic activity (Figure 2A). These results collectively indicate that the presence of a C-terminal lysine decreases potency and infers that other differences in native peptide sequences constitute the basis for higher potency antagonism. Lastly, we investigated the 19C26,A^26^ super-antagonist for its ability to block FGF signaling *in vivo* and found it efficiently inhibits the transcriptional activity of FGF19 and FGF21 in pancreas and adipose, two highly relevant target tissues (Figure 5A,B). Thus, this peptide constitutes the smallest functional antagonist to be reported, and it should become a useful reagent to study *in vivo* physiology of endocrine FGFs.

The wide range of peptide-based antagonistic activities identified through Ala-scan (Table 1) led us to investigate whether comparable alanine mutations in full-length proteins would similarly impact FGF agonism. A single site mutation that selectively destroyed peptide-based antagonism demonstrated an analogous effect to eliminate agonism of full-length proteins. Conversely, a benign substitution for peptide-based antagonism was without impact when introduced into the wild type hormone (Figure 3A). Most importantly, the enhanced KLB binding potency of 19C26,A^26^ translated to a full-length agonist when integrated as a C-terminal substitution in the native FGF21 sequence (FGF21-19A) and yielded a FGF21 analog with increased *in vitro* activity (Figure 3C,D) that was also pharmacologically superior when studied in DIO mice (Figure 5C,D).

Prior models suggest that the terminal ends of the endocrine FGFs individually determine FGFR and KLB recognition, with little or no cross-talk. Our results confirm that short C-terminal sequences are necessary and sufficient to bind KLB and, supportive of a common mechanism, the underlying structural determinants are highly conserved between FGF19 and FGF21. Nevertheless, Lys194 appeared to be a natural molecular break to curb FGF19 agonism (Figure 3C) in an FGFR isoform-specific manner since the FGF19,A^194^ mutant proved about tenfold more effective than FGF19 to signal via FGFR1 but not FGFR4 (Figure 4A,B). This novel finding was further corroborated in a binding assay in which the interaction of FGF19,A^194^ with FGFR1/KLB complex was enhanced but remained unchanged at FGFR4/KLB relative to native hormone (Table 2). Furthermore, the selective ability of the C-terminal lysine in FGF19 to suppress FGFR1-driven signaling relative to FGFR4 indicates that the C-terminal end of the protein operates in concert with the N-terminal region. How this is structurally accomplished remains to be studied, but it illustrates the cooperative action of KLB and FGFR to define high potency of these two endocrine FGFs. As such, the *in vitro* and *in vivo* activities of the engineered FGF21-19A hybrid far exceed those of either native protein (Figure 3, Figure 5D).

In summary, we report here that short C-terminal peptides derived from two endocrine FGFs bind to the KLB co-receptor and can each fully antagonize FGF19 and FGF21 signaling. Despite only a modest degree of sequence identity in the C-terminal regions of FGF19 and FGF21, the critical amino acids involved in KLB recognition are highly conserved between these two proteins. Nonetheless, the differences in sequence can have biological purpose as evidenced by the impact of the FGF19 C-terminal amino acid upon FGFR specificity. As C-terminal lysine residues are susceptible to selective proteolysis by Carboxypeptidase B-like exopeptidases [55], it is plausible that this amino acid in FGF19 might function physiologically to alter receptor selectively. Pragmatically, we believe that the antagonism of the intrinsically derived peptide being subsequently translated to super-agonism when the optimized sequence is integrated as a portion of a full-length protein is precedent setting, as this medicinal chemistry strategy has not been reported before. As well, the synthetic peptide-based KLB antagonist and the super-potent protein hybrid each constitute valuable reagents in further defining the biology of the endocrine FGFs. Collectively, our results deepen the understanding of the structural aspects in FGF19 and FGF21 ligand–receptor complex activation and provide experimental means for further advances in basic and translational research.

## Author contributions

AA, SP, PL, AMKH, and BA designed, performed and analyzed biochemical *in vitro* experiments. AA, JP, and PAM designed, performed and analyzed chemical peptide synthesis. BF and DPT designed, performed and analyzed the *in vivo* experiments. AK and RDD conceptualized, analyzed and supervised all studies. AA, AK, and RDD co-wrote the manuscript.

## Declaration of interests

AA, PL, AK. and RDD are co-inventors on intellectual property at Indiana University and licensed to Novo Nordisk.

## Appendix A Supplementary data

Multimedia component 1 **Table S1**. Nomenclature for FGF protein and C-terminal peptide analogs. **Table S2**. Receptor binding of C-terminal FGF21 Ala-scan peptides. IC_50_ values (μM, mean, n = 2) with fold-change for 21C25 Ala-scan peptides versus native peptide in a cell free AlphaScreen binding assay where the 21C25 Ala peptides competed with FGF21 to associate with the human FGFR1/KLB complex. NC denotes where IC_50_ values were Not Calculated due to negligible activity at the tested concentrations.

Multimedia component 2 **Figure S1. C-terminal FGF peptides dose-dependently antagonize FGF19 and FGF21 activity in 293/KLB cells.** (A) Antagonism of FGF21 by 19C26 (blue), 19C25 (black) and 19C26,ΔT^14^ (red); (B) antagonism of FGF19 by FGF21^18−181^ (black), 21C25 (red), 19C26 (blue) and 19C26,A^26^ (green). The graphs show representative curves plotted by normalizing the pERK signal of each analog with the native peptide's response (A) 19C26 (B) FGF21^18−181^ respectively, mean ± SD, n = 3. **Figure S2. Antagonistic activity of FGF21 and 19 peptides with altered C-terminal residue.** Dose-dependent activity of 21C25 (black), 21C25,K^25^ (red), 19C26 (blue) and 19C26,A^26^ (green) peptides to inhibit (A) FGF21 or (B) FGF19 signaling in Hep3B cells. The graphs show representative curves plotted by normalizing the pERK signal of each analog with the native 21C25 response, mean ± SD, n = 3. **Figure S3. The effects of FGF21 and FGF21-19A hybrid in DIO mice on (A) insulin, (B) fasted plasma glucose, and (C) triglyceride levels.** The measures of each metabolic parameter were taken at the end of the study on day 7 for each treatment dose, FGF21 (red) and FGF21-19A (blue), data were analyzed by 1-way ANOVA with Tukey post-hoc analysis where statistical significance of +P < 0.05 for FGF21 versus FGF21-19A and *P < 0.05 versus vehicle was determined. **Figure S4. Signaling profiles of FGF family members in 293/KLB and 293/KL cells.** Dose-dependent pERK activity measurements of (A) FGF1 (black), FGF1HD (heparin-binding deficient) (red), FGF2 (blue) and FGF21 (green) in 293/KLB cells; (B) FGF1 (black), FGF1HD (red), FGF2 (blue) and FGF23 (green) in 293/KL cells; (C) Biphasic dose response of FGF1 (black) and FGF2 (blue) in 293/KLB cells. The graphs show representative curves plotted by normalizing the pERK signal of each analog with the FGF1 response, mean ± SD, n = 3. **Figure S5. Sequence alignment of select C-terminal sequences of FGF21 and FGF19 representing homology across several species.** (A) FGF21, (B) FGF19 (or FGF15 for mouse): Shaded gray positions display identity, an asterisk (*) indicates positions with a full identity, a colon () indicates positions with changes of conserved properties, a period (.) indicates positions with changes of non-conserved properties; as aligned by Clustal Omega Multiple sequence alignment tool.
