# Supplementary material for: Molecular elements in FGF19 and FGF21 defining KLB/FGFR activity and specificity
Source: Mol Metab. 2018 May 11;13:45–55. doi: 10.1016/j.molmet.2018.05.003 (PMC6026317; doi:10.1016/j.molmet.2018.05.003)
Supplement: Multimedia component 1 — Table S1. Nomenclature for FGF protein and C-terminal peptide analogs. Table S2. Receptor binding of C-terminal FGF21 Ala-scan peptides. IC50 values (μM, mean, n = 2) with fold-change for 21C25 Ala-scan peptides versus native peptide in a cell free AlphaScreen binding assay where the 21C25 Ala peptides competed with FGF21 to associate with the human FGFR1/KLB complex. NC denotes where IC50 values were Not Calculated due to negligible activity at the tested concentrations. [file mmc1.pdf]

SUPPLEMENTARY TABLES

Abbreviation	Sequence definition
21C25	FGF21 157-181 (FGF21 C-terminal 25-amino acid peptide)
19C26	FGF19 169-194 (FGF19 C-terminal 26-amino acid peptide)
$19C26,A^{26}$	FGF19 169-194, K194A
$21C25,K^{25}$	FGF21 157-181, S181K
$19C26,S^{26}$	FGF19 169-194, K194S
$19C26,L^{26}$	FGF19 169-194, K194L
$19C26,E^{26}$	FGF19 169-194, K194E
$19C26,\Delta T^{14}$	FGF19 169-194, des 182T
FGF21	FGF21 1-181
$FGF21^{18\text{-}181}$	FGF21 18-181
$FGF21,A^{164}$	FGF21 1-181, D164A
$FGF21,A^{171}$	FGF21 1-181, P171A
FGF19	FGF19 2-194, P2M
$FGF19,A^{194}$	FGF19 1-194, K194A
FGF21-19A	FGF21 1-156 extended with FGF19 169-194, K194A
FGF1 HD	FGF1 1-140, Q40P, S47I, H93G, K112N, K118E

Table S1.

Position	Residue	Binding	
		IC_{50} (μM)	Fold
21C25		0.15	1.00
157	P	0.42	2.76
158	**P**	0.89	5.92
159	**D**	1.39	9.21
160	**V**	3.98	26.38
161	G	0.16	1.08
162	**S**	2.13	14.13
163	S	0.19	1.27
164	**D**	NC	-
165	**P**	NC	-
166	**L**	NC	-
167	S	0.20	1.34
168	**M**	NC	-
169	**V**	3.11	20.56
170	G	0.18	1.18
171	P	0.13	0.83
172	S	0.12	0.82
173	Q	0.07	0.48
174	G	0.15	1.00
175	R	0.05	0.30
176	**S**	3.14	20.77
177	**P**	2.50	16.58
178	**S**	2.87	18.98
179	**Y**	2.58	17.11
180	A	-	Native
181	S	0.05	0.31

Table S2.
