# Supplementary material for: Molecular elements in FGF19 and FGF21 defining KLB/FGFR activity and specificity
Source: Mol Metab. 2018 May 11;13:45–55. doi: 10.1016/j.molmet.2018.05.003 (PMC6026317; doi:10.1016/j.molmet.2018.05.003)
Supplement: Multimedia component 2 — Figure S1. C-terminal FGF peptides dose-dependently antagonize FGF19 and FGF21 activity in 293/KLB cells. (A) Antagonism of FGF21 by 19C26 (blue), 19C25 (black) and 19C26,ΔT14 (red); (B) antagonism of FGF19 by FGF2118−181 (black), 21C25 (red), 19C26 (blue) and 19C26,A26 (green). The graphs show representative curves plotted by normalizing the pERK signal of each analog with the native peptide's response (A) 19C26 (B) FGF2118−181 respectively, mean ± SD, n = 3. Figure S2. Antagonistic activity of FGF21 and 19 peptides with altered C-terminal residue. Dose-dependent activity of 21C25 (black), 21C25,K25 (red), 19C26 (blue) and 19C26,A26 (green) peptides to inhibit (A) FGF21 or (B) FGF19 signaling in Hep3B cells. The graphs show representative curves plotted by normalizing the pERK signal of each analog with the native 21C25 response, mean ± SD, n = 3. Figure S3. The effects of FGF21 and FGF21-19A hybrid in DIO mice on (A) insulin, (B) fasted plasma glucose, and (C) triglyceride levels. The measures of each metabolic parameter were taken at the end of the study on day 7 for each treatment dose, FGF21 (red) and FGF21-19A (blue), data were analyzed by 1-way ANOVA with Tukey post-hoc analysis where statistical significance of +P < 0.05 for FGF21 versus FGF21-19A and *P < 0.05 versus vehicle was determined. Figure S4. Signaling profiles of FGF family members in 293/KLB and 293/KL cells. Dose-dependent pERK activity measurements of (A) FGF1 (black), FGF1HD (heparin-binding deficient) (red), FGF2 (blue) and FGF21 (green) in 293/KLB cells; (B) FGF1 (black), FGF1HD (red), FGF2 (blue) and FGF23 (green) in 293/KL cells; (C) Biphasic dose response of FGF1 (black) and FGF2 (blue) in 293/KLB cells. The graphs show representative curves plotted by normalizing the pERK signal of each analog with the FGF1 response, mean ± SD, n = 3. Figure S5. Sequence alignment of select C-terminal sequences of FGF21 and FGF19 representing homology across several species. (A) FGF21, (B) FGF19 (or FGF1 [file mmc2.pptx]

## Slide 1
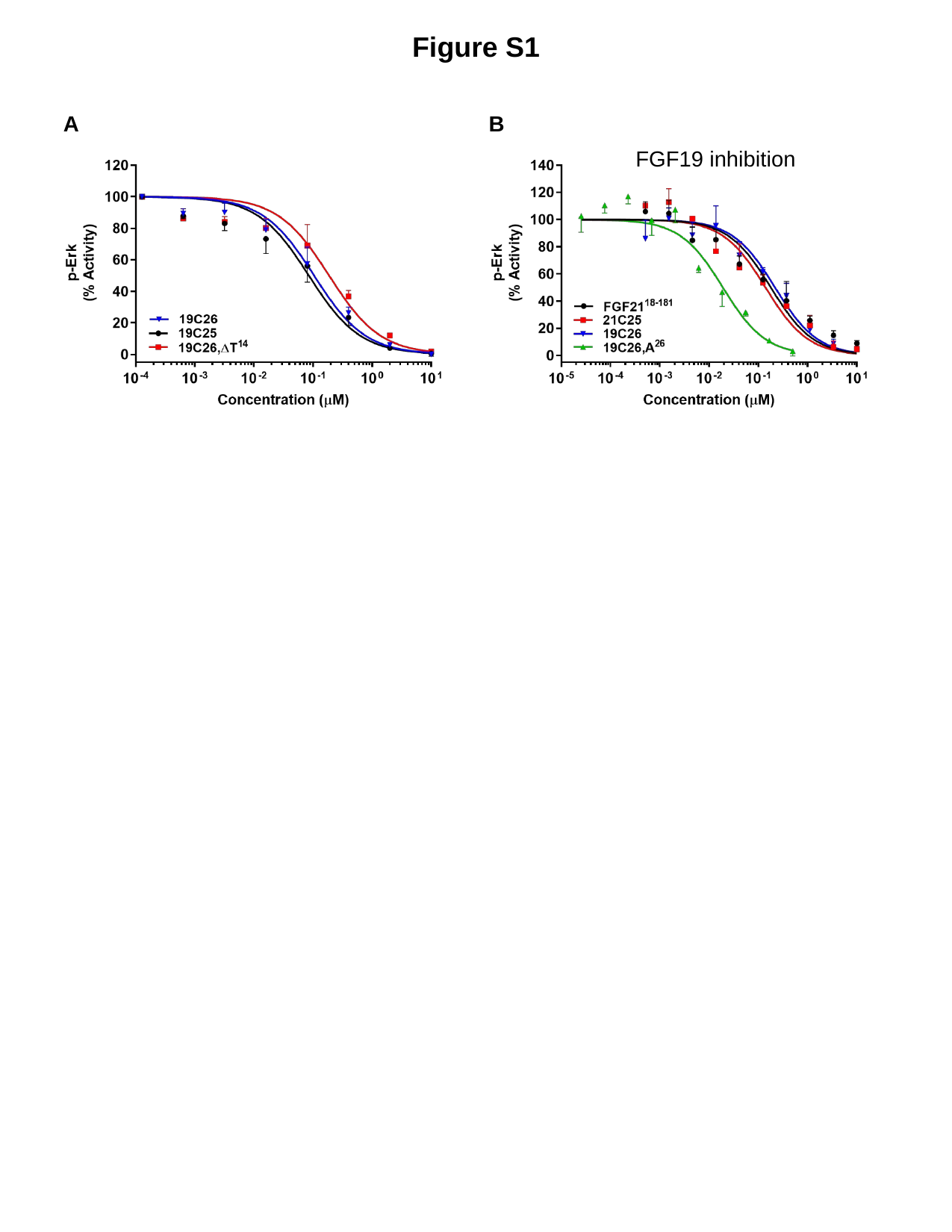

Figure S1
A
B
FGF19 inhibition

## Slide 2
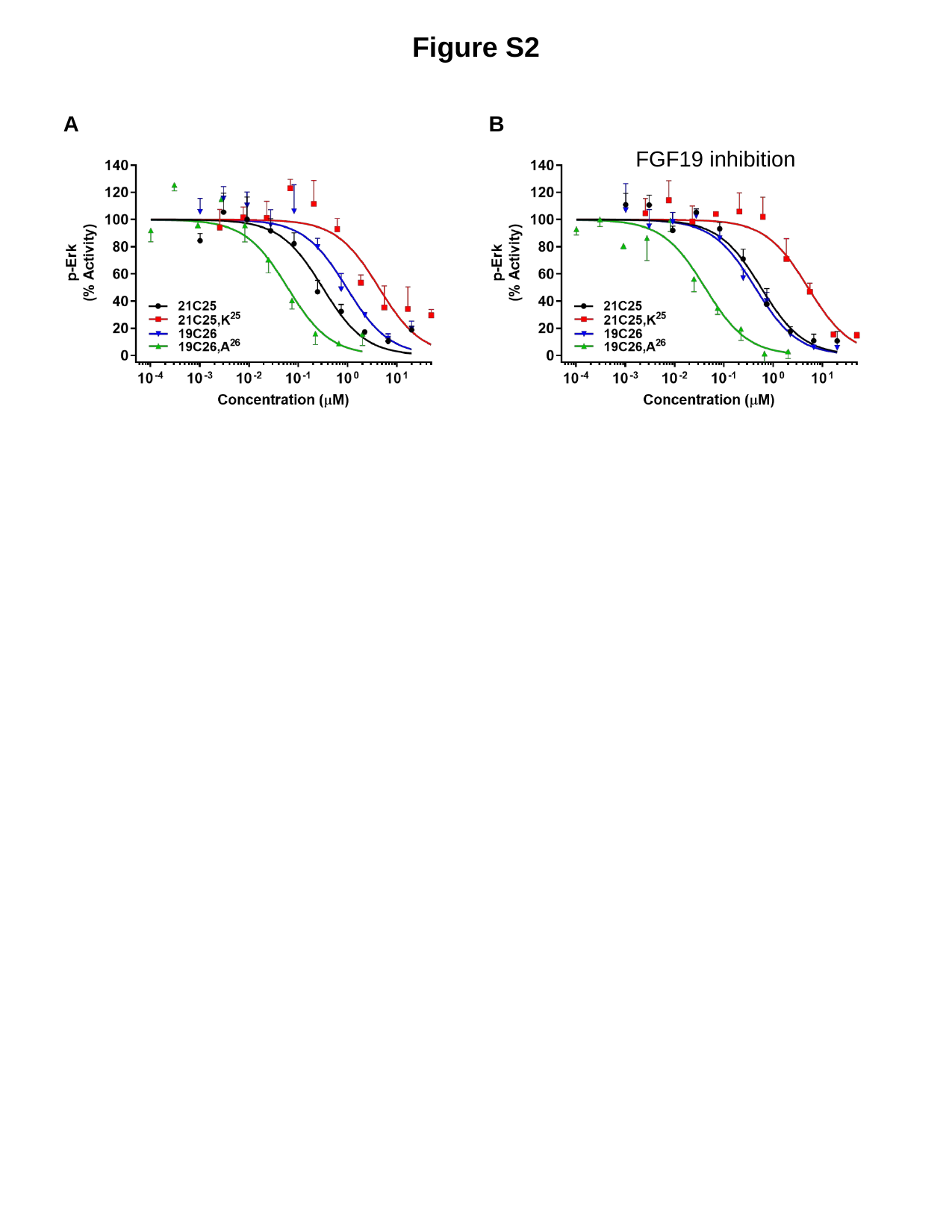

Figure S2
A
B
FGF19 inhibition

## Slide 3
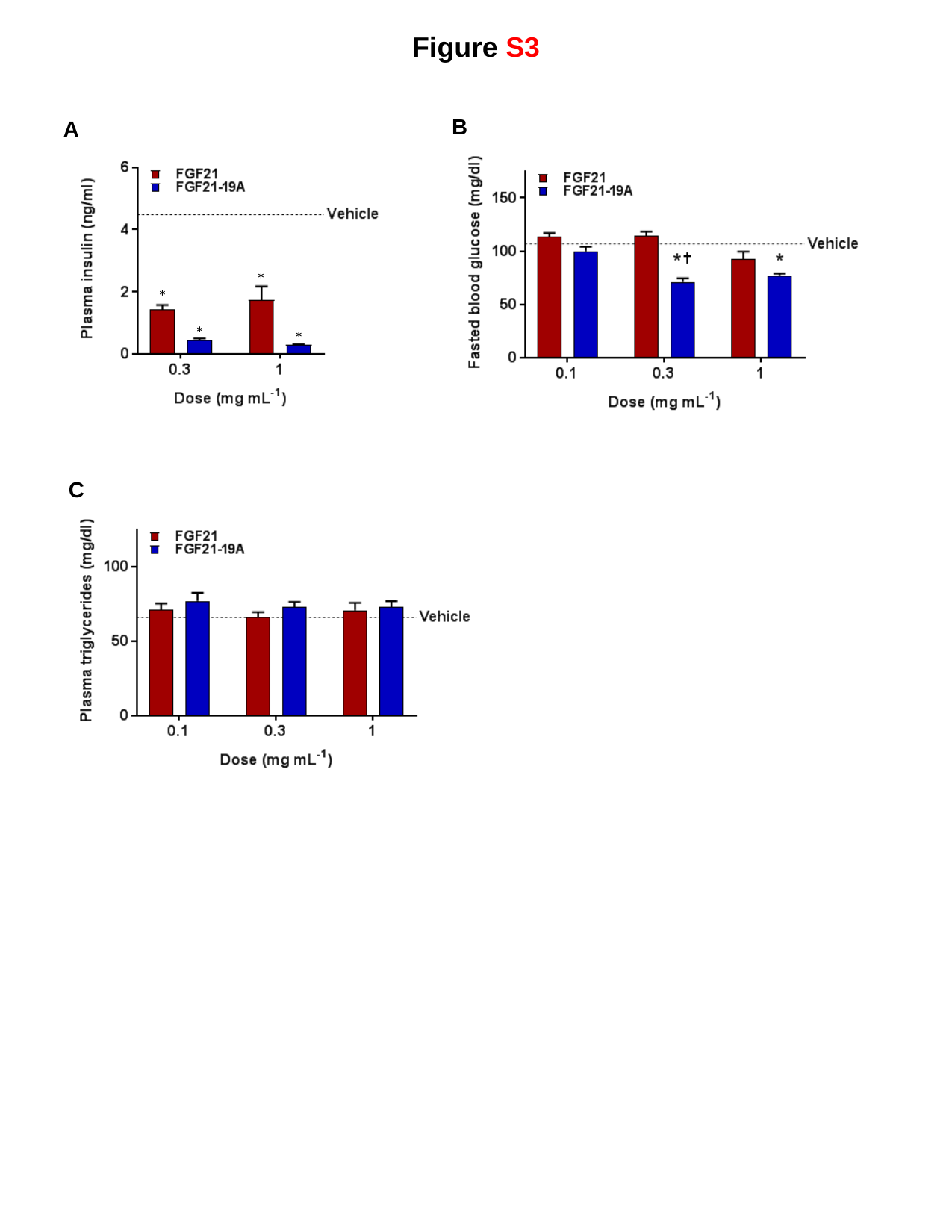

Figure S3
B
A
*
*
*
*
C

## Slide 4
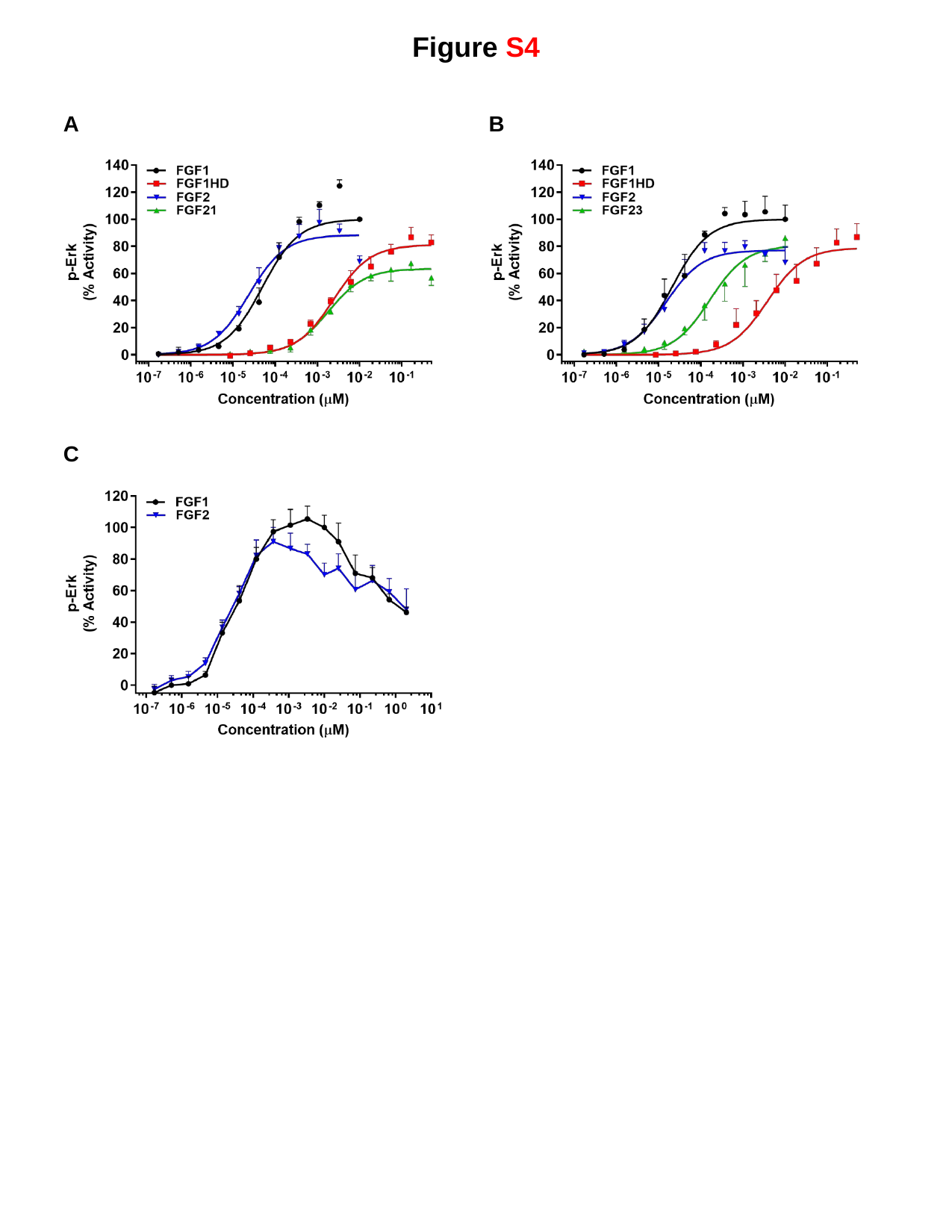

Figure S4
A
B
C

## Slide 5
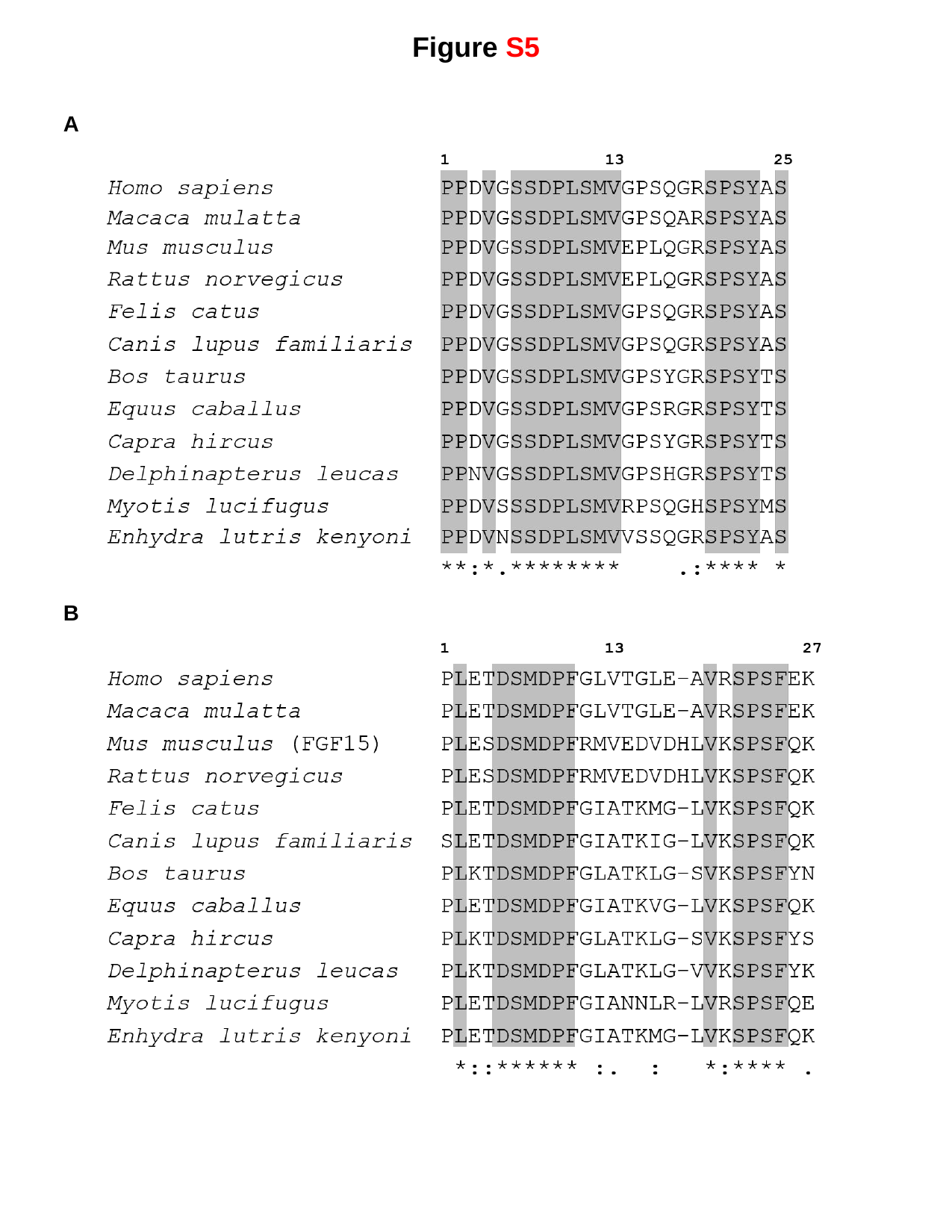

Figure S5
A
B
